# Mitochondrial Voltage-Dependent Anion Channel: From a Passive Pore to a Cellular Hub Through Protein Complexation

**DOI:** 10.3390/ijms27135804

**Published:** 2026-06-26

**Authors:** Megha Rajendran, Sergey M. Bezrukov, Tatiana K. Rostovtseva

**Affiliations:** *Eunice Kennedy Shriver* National Institute of Child Health and Human Development, National Institutes of Health, Bethesda, MD 20892, USA; bezrukos@mail.nih.gov (S.M.B.); rostovtt@mail.nih.gov (T.K.R.)

**Keywords:** VDAC, hexokinase, TOM complex, α-synuclein, tubulin, BCL-2 family proteins, mitochondrial-associated membrane (MAM)

## Abstract

The voltage-dependent anion channel (VDAC) is the primary conduit for ion and metabolite transport across the mitochondrial outer membrane. Positioned at the interface between the cytosol and the mitochondrial compartment, VDAC is uniquely accessible to proteins on both sides of the membrane, making it an interaction hub whose biophysical properties and signaling functions are shaped by protein complexation in addition to its intrinsic pore specialization. Mammals express three isoforms—VDAC1, VDAC2, and VDAC3—sharing a conserved β-barrel scaffold with about 70% identity. However, minor differences in the sequence lead to drastic changes in VDAC isoform affinity with other proteins. Here, we review the molecular mechanisms and physiological consequences of VDAC complexation with a set of well-characterized partners: hexokinase, dimeric tubulin, α-synuclein, mitochondria-associated membrane proteins, B-cell lymphoma 2 (BCL-2) family proteins, and the translocase of the outer membrane (TOM) protein import complex. For each complex, we evaluate the available structural, biophysical, and genetic evidence for isoform specificity, highlight where mechanistic understanding is most advanced, and identify open questions. A consistent principle emerges across all complexes: functionally nonredundant isoform contributions are primarily governed by differential partner affinity and complexation, rather than by differences in pore architecture alone. This framework has direct implications for mitochondria-associated pathologies, including cancer, cardiovascular disease, and neurodegeneration, as well as for the rational design of VDAC-targeting therapeutics.

## 1. Introduction

**Figure 1:**
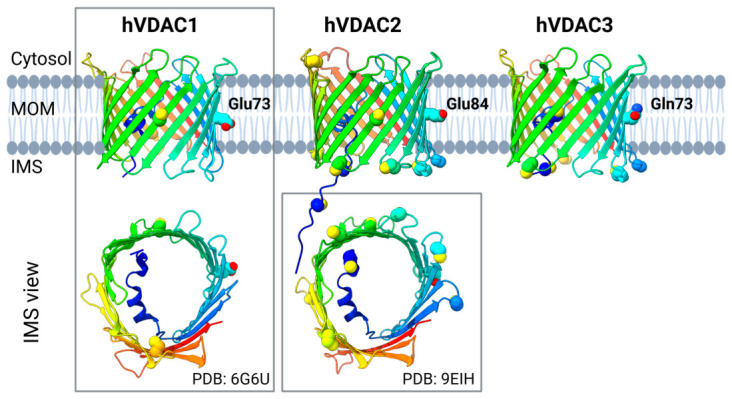
Structural comparison of mammalian VDAC isoforms. Top: Cartoon representation of human VDAC1 (hVDAC1, PDB: 6G6U) derived from X-ray crystallography, colored in rainbow colors with N-terminal in blue and C-terminal in red. Structures of hVDAC2 and hVDAC3 were generated based on sequence homology using ColabFold [8]. Bottom: IMS view of hVDAC1 and hVDAC2, with the latter obtained from cryo-EM of VDAC2-TOM-PINK complex (PDB: 9EIH), shows similar conformations with the N-terminal helix in the lumen of ß-barrel. Isoform-specific differences in cysteine residues and membrane-facing glutamate residues, Glu73 (VDAC1) and Glu84 (VDAC2), and corresponding glutamine (Gln73) in VDAC3 are labeled. (Oxygen: red; nitrogen: blue; sulfur: yellow). Structures derived from X-ray crystallography [9] and cryo-EM data [7] are shown in boxes, along with their corresponding PDB codes. Created on BioRender.

Voltage-dependent anion channels (VDACs) are the principal channels of the mitochondrial outer membrane (MOM) for small-molecule transport. They are among the most abundant integral membrane proteins in the MOM [1]. Localized at the interface between the cytosol and mitochondria, VDACs do more than just conduct water-soluble metabolites and small ions: they also serve as docking platforms for other proteins. Mammals express three VDAC isoforms, VDAC1, VDAC2, and VDAC3, which share a conserved pore-forming scaffold but differ in sequence features that are likely responsible for the differences in partner recognition and regulation. Comparative analyses highlight an extended N-terminus in VDAC2 and marked differences in cysteine content and distribution among the three isoforms, despite their overall conservation [2,3] (Figure 1). Only the structure of VDAC1 has been solved [4,5,6]; however, sequence homology and computational studies suggest a largely conserved three-dimensional framework (Figure 1). Recently, a cryo-electron microscopy (EM) study of the VDAC2 complex with translocase of the outer membrane 40 (TOM40) and PTEN-induced kinase 1 (PINK1) confirmed the ß-barrel structure of VDAC2 with the N-terminus extending into the intermembrane space (IMS) [7] (Figure 1).

Since the discovery of VDAC in 1976 [10,11,12] by Colombini, Mannella, and coauthors, for another 20 years, this mitochondrial channel was conventionally regarded as a simple molecular sieve. Only in the mid-90s was VDAC experimentally shown to translocate ATP by passive diffusion and to regulate ATP flux by voltage-gating [13]. Since then, VDAC is increasingly viewed not as a passive β-barrel mitochondrial channel, but as a regulated hub whose permeability and signaling functions are shaped by interactions with other proteins. The unique VDAC location at the intersection between mitochondria and cytosol allows VDAC to regulate communication between mitochondria and the rest of the cell by complexing with a plethora of proteins involved in cell signaling and metabolism [14,15,16]. This view is supported by both targeted studies and broader interactome approaches. Roman et al. used a membrane-protein-adapted phage display strategy to identify many previously known ligands and numerous additional VDAC-interacting epitopes [17]. For instance, VDAC was found to be associated with different pro- and anti-apoptotic proteins [18,19,20,21,22], multiple kinases [23,24,25,26,27,28], cytoskeleton [29,30,31], neuronal [32,33,34], and mitochondrial proteins [7,35]. In addition, distinct protein-partner repertoires provide a mechanistic basis for the nonredundant contributions of VDAC1, VDAC2, and VDAC3 to metabolism, apoptosis, redox regulation, and inter-organelle communication [36]. Thus, the strongest current framework for understanding isoform-specific VDAC biology is not only through its channel properties but also differential complexation with protein partners.

This review approaches VDAC primarily as an interaction hub and treats isoform-specific complexation as the key to understanding how structurally similar ß-barrel mitochondrial channels acquire distinct cellular functions.

## 2. Hexokinase–VDAC Complex

VDAC complexation with hexokinase (HK) at the MOM links mitochondrial metabolism to glycolysis [37]. Early work showed that a substantial fraction of brain HK is associated with particulate matter, which was identified as the mitochondrial fraction [38,39]. Felgner and colleagues extended this finding by purifying an HK-binding protein from MOM, which was later identified as VDAC [26,40,41].

**Figure 2:**
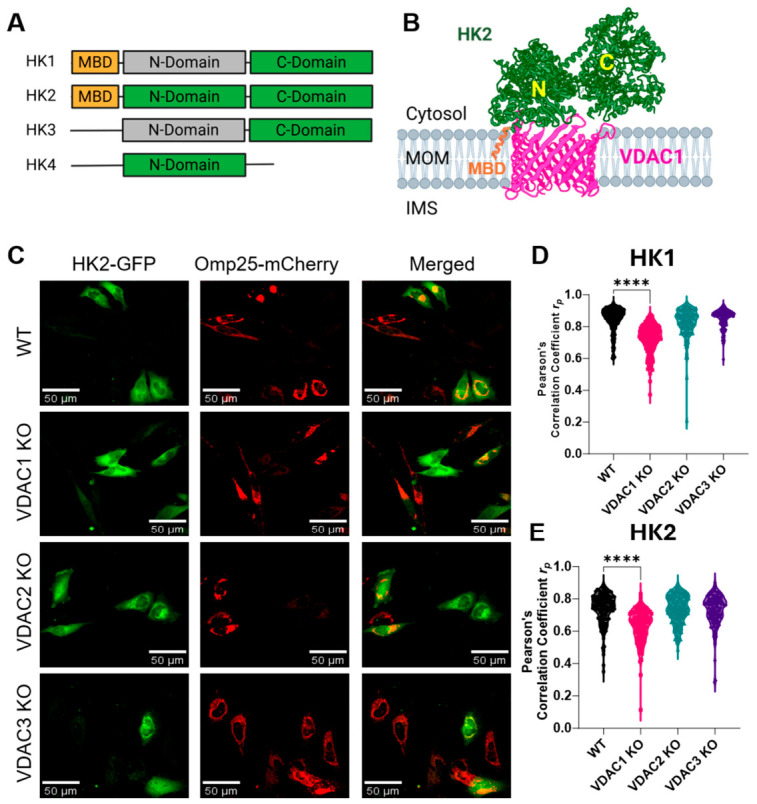
VDAC complexation with hexokinase (HK). (**A**) Schematic representation of the four major HK isoforms with the mitochondrial binding domain (MBD) shown in orange for HK1 and HK2. Except HK4, all isoforms have two domains, with the catalytically active domain shown in green. (**B**) Putative VDAC-HK complexation after Haloi et al. [50]. HK MBD inserts into MOM and interacts with VDAC1 at the membrane interface. (Created on BioRender) (**C**) Colocalization of HK2-GFP (green) and Omp25-mCherry (red) shown in the merged image for HeLa WT, VDAC1, 2, and 3 KO cell lines. Pearson’s Correlation Coefficient analysis of HK1 (**D**) and HK2 (**E**) colocalization with Omp25-mCherry reveals a significant decrease in mitochondrial localization of HK1 and HK2 in VDAC1 KO cell lines. Significance was tested using one-way ANOVA followed by Dunnett’s multiple comparison test (**** *p* < 0.0001) (**C**–**E**) were adapted with permission from [49].

Around the same time, Polakis and Wilson showed that the hydrophobic N-terminal sequence of rat brain HK is required for mitochondrial targeting [42]. There are four major HK isoforms, but only HK1 and HK2 have an N-terminal membrane-binding domain that associates with the MOM (Figure 2A) [37]. HK2-specific mutational analysis further confirmed that residues within the N-terminus are critical for mitochondrial binding, with substitution of His5 abolishing outer-membrane association [43]. While the N-terminal peptide is necessary for mitochondrial targeting, the N-terminal binding domain may not be sufficient since fusion experiments showed that the HK1 binding domain could not target HK4 to mitochondria, indicating that adjacent structural elements also contribute to docking competence [44]. Additional work arguing for multiple mitochondrial HK-binding modes or sites, including distinctions between glucose-6-phosphate-sensitive and -insensitive interactions, also suggests that HK isoform specificity may intersect with binding heterogeneity rather than reflecting a single uniform docking mechanism [45,46].

VDAC isoform specificity further complicates our understanding of HK-VDAC complexation. Earlier work often treated VDAC as a single receptor entity, but later studies began to resolve the interaction more explicitly at the isoform level. Deletion of VDAC1 reduced mitochondria-bound HK activity in oxidative tissues such as heart and soleus muscle and is associated with impaired glucose tolerance, supporting VDAC1 as a physiologically important mitochondrial HK-binding site in vivo [47]. Later cell-based work further showed that VDAC1 loss reduces mitochondrial HK2 binding under oxidative stress conditions in the rat myoblast cell line (H9c2), extending the relevance of the interaction beyond HK1 alone [48]. More recent comparative analysis by our group strengthened the VDAC1-centered view in HeLa cells [49]. VDAC1 knockout (KO) decreased glycolysis rate, whereas VDAC2 and VDAC3 KO did not produce the same glycolytic defect, linking VDAC1 most directly to glycolytic support in HeLa cells. We also showed that mitochondrial localization of both HK1-GFP and HK2-GFP, measured by colocalization with the outer-membrane marker Omp25-mCherry (Figure 2C–E), is significantly reduced in VDAC1 KO cells, further supporting a privileged role for VDAC1 in recruiting or stabilizing mitochondrial HK in this cellular context (Figure 2B) [49]. Using molecular dynamics (MD) simulations, Haloi et al. proposed a membrane-first model in which HK2 initially binds the MOM via its N-terminal segment and then engages VDAC1 at the membrane interface [50]. Recently, Bieker et al. suggested that HK1 binds VDAC1 and VDAC2 through the interaction of the N-terminal of HK1 with the membrane-facing glutamate in VDAC1 (Glu73) and VDAC2 (Glu84) [51]. The dissociation of HK1 from mitochondria upon expression of VDAC1 and VDAC2, with the glutamate residue mutated, was used as evidence for direct binding of the HK1 N-terminus to the membrane-facing glutamate residue. However, mutating Glu73 may disrupt other protein–protein interactions and cellular processes, which may indirectly affect HK1 binding. In addition, HK1 showed a higher degree of colocalization with VDAC3, the isoform lacking Glu73 but carrying neutral Gln73, at the spatial resolution achieved by two-color STED microscopy. A substantial fraction of mitochondria-bound HK1 did not colocalize with any of the three isoforms, suggesting a more complex spatial relationship than previously anticipated [52]. Therefore, future work is needed to resolve the structure of the HK complex with each VDAC isoform.

Historically, mitochondrially bound HK was suggested to preferentially phosphorylate glucose using intramitochondrially generated ATP in hepatoma mitochondria, providing direct evidence that the VDAC-HK complex can channel ATP from oxidative phosphorylation into the first step of glycolysis [37,53]—the so-called channeling model. More recently, Huggler et al. reported that cytosolic HK, rather than outer-membrane-docked HK, better supports aerobic glycolysis and cell growth in their system, directly challenging the broadest version of the classic channeling model [54]. The VDAC-HK complex has also been suggested to act as a “battery” leading to metabolic regulation of MOM potential and therefore permeability for metabolites through VDAC regulation by voltage [55]. Thus, the controversy in the field is no longer whether HK can bind VDAC, but how general the metabolic consequences of that binding are and whether channeling models apply uniformly across systems.

A separate line of work has linked HK-VDAC to cell death control, but the protective role of a defined HK-VDAC complex remains incompletely established. Perturbation studies show that disrupting the HK2-VDAC1 axis increases susceptibility to mitochondrial injury under stress conditions [23,48] and can also promote inflammasome signaling [56] or stress-induced mitochondrial remodeling [57], yet these results remain largely indirect and do not by themselves demonstrate that HK-VDAC binding is the primary protective determinant against apoptosis.

## 3. Tubulin–VDAC Complex

Beyond its canonical role as the building block of microtubules, free dimeric tubulin also associates with mitochondria. Early evidence came from Bernier-Valentin et al., who demonstrated high-affinity tubulin binding to purified mitochondrial membranes [58], and from Saetersdal et al., who used immunofluorescence and immunogold EM to show β-tubulin association with MOM in adult cardiac myocytes [59]. Carré et al. later provided direct biochemical evidence for a VDAC–tubulin complex at MOM, showing that tubulin is an inherent component of mitochondrial membranes and accounts for ~2% of total cellular tubulin. It is likely organized as an α/β heterodimer, enriched in βIII-tubulin, and specifically co-immunoprecipitates with VDAC [60]. However, the physiological role of free cytosol-solubilized dimeric tubulin, other than being a supply for microtubules, was unknown until recently.

**Figure 3:**
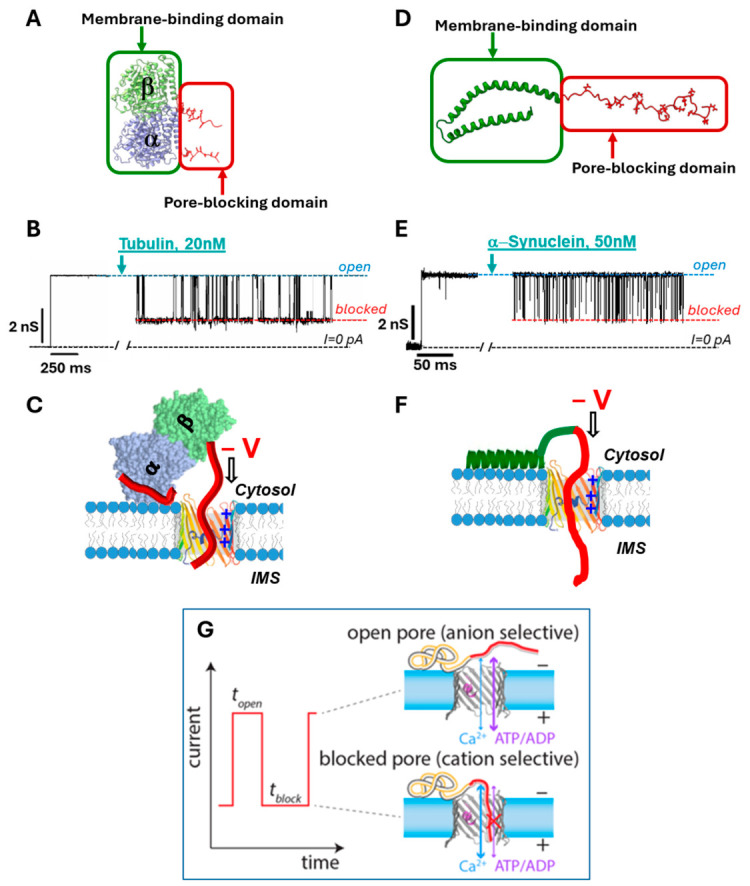
VDAC reconstitution experiments. Dimeric tubulin (**A**–**C**) and αSyn (**D**–**F**) induce fast, transient, partial blockages of VDAC conductance. (**A**,**D**) Both VDAC inhibitors, tubulin and αSyn, have two domains: a membrane binding domain (green frame), which can be structured or not, and a disordered polyanionic pore-blocking domain (red frame). Records of fluctuating conductance of single VDAC channels reconstituted into PLM before (left traces) and after addition of 20 nM tubulin (**B**) or 50 nM αSyn (**E**) to one side of the membrane at −25 mV applied voltage. Potential is more negative at the side of tubulin or αSyn addition. The channel conductance fluctuates between the open and blocked states, as indicated by the blue and red dashed lines, respectively, with well-time-resolved blockage events. Long dashed black lines indicate the zero-current corresponding to negligible conductance. Models of VDAC complexation with dimeric tubulin (**C**) and αSyn (**F**), in which the negatively charged C-terminal tails (CTT, shown in red) of either tubulin or αSyn, driven into the VDAC pore by the applied negative potential, partially block the channel. The relatively short α- and the longer β-tubulin CTT (**A**) can be captured by the pore only when the α-tubulin subunit is membrane-bound [69]. Due to its bulkiness, tubulin does not translocate through VDAC. The membrane-bound N-terminal domain of αSyn (**D**) prevents translocation of the whole molecule through the pore only up to a certain voltage [32,70]. (**G**) Each interaction of blocker with channel is characterized by the open time (t_open_) and the blockage time (t_block_). The open state is anion-selective, allowing ATP/ADP fluxes while exhibiting limited Ca^2+^ permeability. The blocked state is cation-selective and impermeable to ATP/ADP, but it has higher permeability for Ca^2+^. Adapted with permission from [66].

Dimeric tubulin is a heterodimer of around 110 kDa with α- and ß-subunits with well-defined structures of their globular parts [61]. Each tubulin subunit also has an unstructured, highly negatively charged C-terminal tail (CTT) composed of 10–27 amino acids [62] that are essentially poly-glutamate polypeptides exposed at the protein surface (Figure 3A). Higher eukaryotes encode six to nine tubulin isotypes of each subunit with cell-specific expression [63]. Notably, the seven α-tubulin and eight ß-tubulin known isotypes in humans vary mostly in their CTTs [64].

Our group showed that dimeric tubulin interacts with VDAC reconstituted into planar lipid membrane (PLM) and induces characteristic fast reversible blockages of VDAC conductance on the 1 ms to 1000 ms time scale [30,65]. A representative experiment in which 20 nM of dimeric tubulin isolated from the brain induces fluctuations in channel conductance between high conducting “open” and low conducting “blocked” states is shown in Figure 3B. Dimeric tubulin induces measurable blockage events only when negative voltage is applied to the side of tubulin addition (Figure 3C). This observation and the fact that tubulin with proteolytically cleaved CTT does not induce channel blockages lead to the conclusion that anionic CTTs are responsible for pore blockage [30]. The blockage time—the time when the channel is blocked by tubulin—is highly voltage dependent and increases exponentially with applied voltage [30,65,66]. Another piece of experimental evidence that the anionic CTT of tubulin is captured inside the VDAC pore, driven there by negative voltage, is that the tubulin-blocked state has reversed, cationic selectivity, in contrast to the anionic selectivity of the open state [67]. Equilibrium and nonequilibrium MD simulations, together with the Rosetta protein–protein docking algorithm, provided further structural evidence of the tubulin–VDAC complex with the anionic CTT captured inside the pore and reversing its selectivity [68]. Another important feature of the tubulin–VDAC interaction is that the CTT alone is not sufficient to produce long-lived VDAC blockages. CTT should be attached to an “anchor” that localizes CTT near the channel entrance and prevents its free translocation through the pore (Figure 3C).

Therefore, the first step of the tubulin–VDAC interaction is tubulin binding to the lipid surface, as confirmed by several biophysical methods and MD simulations [69]. Furthermore, using a recombinant yeast tubulin construct with either α- or ß-tubulin CTT in experiments with reconstituted VDAC, we showed that both α- and ß-CTTs could be captured by the VDAC pore [71]. Taken together, these data allowed us to propose a model of the tubulin–VDAC interaction where anionic tubulin CTT partially and transiently blocks the positively charged VDAC pore. This causes reversal of its ionic selectivity, and complete blocking of ATP transport through the pore while still allowing for transport of small ions, including Ca^2+^ [66,67,68] (Figure 3C).

The most important physiological consequence of VDAC’s reversible blockage by tubulin CTTs for mitochondria is that the tubulin-blocked state of the channel is essentially impermeable for ATP, as has been shown by direct experiments on reconstituted channels [67] and MD simulations [68]. Thus, tubulin can effectively regulate the fluxes of metabolites (most of which are negatively charged) through VDAC and, consequently, MOM permeability and mitochondria respiration. Indeed, these predictions were confirmed in experiments with isolated mitochondria [30,72] and intact cancer cells [73,74]. It was shown that when a cytosolic pool of free dimeric tubulin in human hepatoma (HepG2) cells was increased after the treatment with the microtubule-targeting drugs (MTD) causing microtubule destabilization (such as nocodazole), the mitochondrial potential decreased [73]. In contrast, treatment with a microtubule-stabilizing agent (such as paclitaxel) decreased free cytosolic tubulin and led to a rise in mitochondrial potential [73]. The subsequent genetic evidence of VDAC’s involvement in the modulation of mitochondrial potential by MTDs was obtained using the siRNA knockdown of each VDAC isoform in HepG2 cells [74]. These experiments unambiguously showed that knockdown of each VDAC isoform led to an isoform-dependent decrease in mitochondrial potential. The observed changes in mitochondrial potential were suggested to be caused by a decrease in ATP/ADP uptake and an increase in Ca^2+^ uptake by VDAC when blocked by free dimeric tubulin. The close proximity of ßIII-tubulin to VDAC1 was later confirmed using a proximity ligation assay (PLA) in neuroblastoma cells [71].

Together, these findings support a model of the tubulin–VDAC complex, which dynamically modulates VDAC permeability at the MOM, thereby regulating metabolite fluxes in and out of mitochondria, mitochondrial membrane potential, and respiration. More recent work by Parker et al. further links βIII-tubulin to mitochondrial biology. Parker et al. showed that βIII-tubulin promotes a more extended reticular mitochondrial network, whereas βIII-tubulin suppression shifts mitochondria toward a more perinuclear distribution [75], and that βIII-tubulin alters cellular metabolism and stress-response signaling under glucose limitation [76].

## 4. α-Synuclein–VDAC Complex

α-Synuclein (αSyn) is a small, 140-residue, 14.4 kDa intrinsically disordered protein that is highly expressed in neurons, particularly at presynaptic terminals of neurons [77]. It is central to Parkinson’s disease (PD) pathology [78]. αSyn is a major constituent of Lewy bodies, and *SNCA* point mutations, multiplications, and regulatory variants link altered αSyn sequence or dosage to familial and sporadic synucleinopathies [79]. αSyn toxicity is often discussed in the context of its oligomer and fibril formation [78], but the detailed mechanisms by which αSyn is involved in neurodegeneration, particularly in its monomeric form, remain largely unresolved.

Early studies showed that a fraction of αSyn localizes to both outer and inner mitochondrial membranes [80,81], causing impairment of the mitochondrial respiratory complexes I [82,83], IV [84], and V [85], increasing oxidative stress [86,87] and fission [88] and leading to neuronal cell death (for the recent review, see [89,90]). More direct evidence for a VDAC-centered pathway came from neuronally differentiated SH-SY5Y cells overexpressing wild-type αSyn, where PLA detected αSyn in close proximity to both VDAC1 at the outer membrane and COX IV at the inner membrane; importantly, VDAC1 knockdown markedly reduced αSyn proximity to COX IV [91], supporting VDAC as a leading route for αSyn passage across the outer membrane.

Mechanistically, αSyn resembles tubulin in combining a membrane-binding domain with a highly acidic, disordered CTT capable of pore engagement (Figure 3D). Structural studies show that upon binding to an anionic membrane, the N-terminal region of αSyn becomes α-helical and acts as a membrane anchor, whereas the C-terminal region remains disordered and only weakly associated with the membrane [92,93], thus leaving it available for interaction with VDAC. Consistent with this model, the biophysical work demonstrated that monomeric αSyn at nanomolar concentrations reversibly blocks reconstituted VDAC in planar bilayers [32] (Figure 3E). The single-channel recordings show markedly similar fluctuations between the open VDAC state and the blocked state, the latter with the conductance ~0.4 of the open state conductance, in the presence of either αSyn or tubulin (Figure 3B,E). Importantly, similar to tubulin, increasing the applied negative voltage keeps the anionic CTT of αSyn inside the pore longer, which is seen as an exponential increase in the blockage time, and selectivity of the αSyn blocked state shifts from anionic to cationic [70,94] (Figure 3F,G). At relatively low voltages, the pore-blocking domain—CTT of αSyn or tubulin—is retracted back to the membrane surface after some time spent inside the pore. At higher voltages, the αSyn blockage time decreases, consistent with a transition from reversible C-terminal capture to αSyn translocation through the pore [32,70]. The translocation regime for αSyn was demonstrated in experiments on VDAC reconstituted in PLM in a salt gradient, using direct single-channel, real-time monitoring of αSyn translocation through the pore [70]. Evidently, tubulin, whose bulky body prohibits its translocation through the pore, does not show a reduction in blockage time even at voltages higher than 40 mV. This framework suggests several consequences for mitochondrial physiology. First, αSyn may regulate MOM permeability by dynamically restricting VDAC-mediated flux of ATP/ADP and other anionic respiratory substrates. Second, despite lowering overall conductance, the αSyn-blocked state strongly favors Ca^2+^ flux, offering a mechanistic link to reports that αSyn expression enhances mitochondrial Ca^2+^ uptake [95] (Figure 3G). Third, under conditions of αSyn overexpression or stress, VDAC-mediated translocation provides a plausible route by which cytosolic αSyn reaches intramitochondrial targets and contributes to respiratory dysfunction and loss of mitochondrial membrane potential. Taken together, these observations broaden the discussion beyond the toxic role of aggregated αSyn and support a model in which membrane-bound monomeric αSyn directly modulates VDAC function and mitochondrial metabolism in both healthy and pathological conditions.

A further layer of complexity is introduced by VDAC isoform specificity. Single-channel studies demonstrated that αSyn can block and translocate through all three mammalian VDAC isoforms, but with quantitatively distinct kinetics: VDAC3 displays 10- to 100-fold lower on-rates for αSyn-induced block than VDAC1 [96], and the VDAC2-αSyn interaction is ~ 10 times less effective than that of VDAC1-αSyn [97]. This finding supports the idea that differential affinity for cytosolic disordered proteins may contribute to nonredundant isoform function in cells, although the physiological consequences of these kinetic differences still need to be defined more directly.

## 5. VDAC-Mitochondria-Associated Membrane Protein Complexes

Close contact sites between mitochondria and other organelles, such as endoplasmic reticulum (ER), sarcoplasmic reticulum (SR), and lysosomes, known as mitochondria-associated membranes (MAMs), have been implicated in sterol homeostasis, Ca^2+^ signaling, and ER stress pathways.

**Figure 4:**
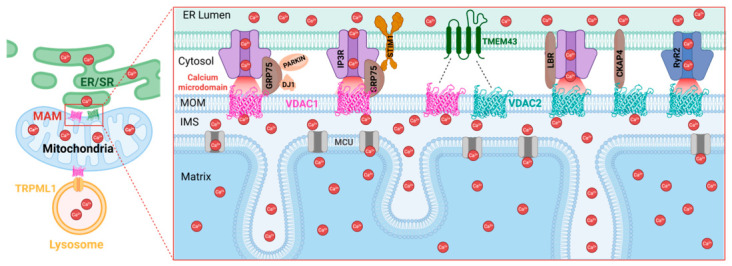
VDAC complexation at mitochondria-associated membranes (MAMs). Cartoon representation of VDAC complexes at the contact sites with other organelles such as endoplasmic reticulum (ER), sarcoplasmic reticulum (SR), and lysosome (cartoon at the left). VDAC1 has been shown to form complexes with Ca^2+^ transporter TRPML1 at the lysosome and with multiple proteins at the ER/SR contact sites (zoomed red box). VDAC1 complex with ER Ca^2+^ transporter IP3R through GRP75 allow rapid Ca^2+^ uptake from ER to mitochondria via the formation of Ca^2+^ microdomains at the interface. This complex can be modulated by Parkinson’s disease-related proteins, such as PARKIN and DJ1, and is linked to ER stress. STIM1, an ER Ca^2+^ sensor, promotes ER-mitochondria Ca^2+^ flux through interaction with the IP3R-GRP75-VDAC1 complex. TMEM43, an ER/nuclear protein, was shown to promote ER–mitochondria contact sites through interaction with VDAC1 and VDAC2. Lamin B receptor (LBR) promotes Ca^2+^ flux through the IP3R-VDAC2 complex, while CKAP4 competes with IP3R for binding VDAC2, reducing the IP3R-VDAC2 interaction. VDAC2 also forms a complex with RyR2 in SR. Created on BioRender.

Ca^2+^ acts as a secondary signaling molecule to coordinate cell activity with mitochondrial ATP production. This requires rapid Ca^2+^ uptake into the mitochondrial matrix by mitochondrial Ca^2+^ uniporters (MCUs) located at the inner membrane. However, MCUs have low affinity for Ca^2+^ [98] compared to the speed of Ca^2+^ accumulation observed in live cells. MAMs link VDAC to other Ca^2+^ channels, such as inositol-1,4,5-triphosphate-sensitive channels (IP3R) of ER [99], Ryanodine-sensitive channels (RYRs) of SR [100], and transient receptor potential mucolipin 1 (TRPML1) of lysosomes [101] (Figure 4). These Ca^2+^ microdomains facilitate rapid Ca^2+^ uptake via VDAC and MCU.

Early work established VDAC as an important part of Ca^2+^ complexes at contact sites to promote the transfer of local ER Ca^2+^ signals to mitochondria [102]. Later, VDAC1 was identified as a core component of the IP3R-GRP75-VDAC1 axis [103,104]. In particular, despite all three VDAC isoforms being involved in mitochondrial Ca^2+^ uptake upon IP3R stimulation [105], only VDAC1 was reported to form complexes with IP3R and to selectively support transmission of low-amplitude apoptotic Ca^2+^ signals to mitochondria. However, more recent studies re-established the role of VDAC2 in Ca^2+^ signaling. Harada et al. identified palmitoylated CKAP4 as a VDAC2-binding partner at ER–mitochondria contact sites that restrains IP3R–VDAC2 coupling and mitochondrial Ca^2+^ loading [106], whereas Zhao et al. showed that the lamin B receptor (LBR) organizes a mitosis-specific IP3R–LBR–VDAC2 contact that enhances mitochondrial Ca^2+^ influx to support ATP production and cell division [107]. Furthermore, the RyR2-VDAC2 association at SR–mitochondria junctions has been linked directly to mitochondrial Ca^2+^ uptake in cardiomyocytes [108]. Cardiac-specific deletion of VDAC2 caused impaired mitochondrial Ca^2+^ uptake, altered intracellular Ca^2+^ transients, defective excitation–contraction coupling, and dilated cardiomyopathy, indicating that VDAC2 has a nonredundant role in maintaining mitochondrial Ca^2+^ handling in the heart [109].

Comparative cardiac experiments showed that VDAC3 is less effective than VDAC1 or VDAC2 in supporting mitochondrial Ca^2+^ transfer, and isoform-specific mutational analysis pointed specifically to the membrane-facing glutamate, showing that the native Gln73 in VDAC3 contributes to its weaker support of mitochondrial Ca^2+^ transfer relative to the glutamate found at the corresponding position in VDAC1 and VDAC2 [110]. However, considering that the Ca^2+^ ion cannot reach membrane-facing glutamate through the hydrophobic lipid core (Figure 1), the mechanism by which Glu73 regulates mitochondrial Ca^2+^ uptake in HL-1 cardiomyocytes remains unknown. We can only speculate that, in cardiac cells, there is an ER Ca^2+^ channel that forms a putative complex with the VDAC2 hydrophobic exterior via Glu84.

Beyond mitochondrial Ca^2+^ flux itself, MAM-associated VDAC complexes are increasingly implicated in ER stress pathways. ER stress activates the unfolded protein response (UPR) and ER-associated degradation (ERAD) to restore proteostasis, but prolonged stress can remodel ER–mitochondria contact sites, where altered Ca^2+^ transfer can amplify mitochondrial dysfunction. In this context, the classical IP3R–GRP75–VDAC1 complex is increasingly understood not simply as a Ca^2+^ conduit, but as an ER stress signaling hub. Meng et al. identified a STIM1-regulated IP3R–GRP75–VDAC1–MCU axis. STIM1, an ER Ca^2+^ sensor, promotes assembly or stabilization of the ER–mitochondria Ca^2+^-transport complex, thereby enhancing mitochondrial Ca^2+^ loading, oxidative stress, and downstream injury during inflammation [111]. This idea was reinforced by Yan et al., who showed that an ERAD modulator (NCATS-SM0225) binds all three VDAC isoforms, strengthens VDAC1–IP3R coupling, and activates UPR response to selectively kill cancer cells [112]. Along with this model, Orantos-Aguilera et al. showed that STIM1-containing contact sites also promote direct ER-to-mitochondria Ca^2+^ flux through GRP75- and PTPIP51-associated MAMs, extending the classical IP3R–VDAC1 framework to a broader contact-site model for mitochondrial Ca^2+^ uptake [113]. In cancer, chronic ER stress can drive a more adaptive UPR/ERAD program that helps sustain survival and metabolic remodeling. In contrast, ERAD is more often linked to the accumulation of misfolded proteins in neurodegeneration. Two PD-linked proteins, Parkin and DJ-1, regulate VDAC1-centered ER–mitochondria contact: Xue et al. showed that Parkin modulates the IP3R–GRP75–VDAC1 complex to influence ER–mitochondria association and Ca^2+^ homeostasis [114], whereas Liu et al. identified DJ-1 as a stabilizing partner of the IP3R3–GRP75–VDAC1 complex [115]. These studies suggest that disruption of VDAC1-centered MAM signaling may be one route by which PD-associated genes perturb mitochondrial homeostasis under stress.

TMEM43/*LUMA*—an ER and nuclear-envelope membrane protein [116,117]—has also been identified as a component of an ER-membrane E3 ubiquitin ligase (RNF26) complex, linking TMEM43 more broadly to the ERAD pathway [116,117,118]. Mutations in this protein are associated with muscular dystrophy [119], arrhythmogenic right ventricular cardiomyopathy [120], and cancer [121]. TMEM43/*LUMA* has been linked more broadly to cardiac and metabolic regulatory networks, as systems-genetics analysis and *Tmem43*-S358L mouse studies have associated altered TMEM43 with cardiomyopathy- and mitochondria-related pathways [122]. In particular, the *TMEM43*p.S358L mutant weakens the interaction with VDAC1 and VDAC2 and destabilizes ER/SR–mitochondria contact sites, leading to impaired mitochondrial function and cardiac cell death [123,124]. Zhang et al. showed that TMEM43 can promote hepatocellular carcinoma by enhancing VDAC1 activity through Ubiquitin-specific-processing protease 7 (USP7)-dependent deubiquitination, suggesting that TMEM43–VDAC coupling can also be co-opted to support metabolic adaptation and survival in cancer [125]. Together, these studies suggest that ER stress phenotypes can arise not only from altered Ca^2+^ flux through canonical IP3R–VDAC complexes, but also from disruption of the contact-site scaffolds that organize those complexes.

## 6. VDAC-BCL-2 Family Protein Complexes

Apoptosis, a complex cell death pathway, often leads to MOM permeabilization (MOMP), driven by the oligomerization of the effector proteins such as BAX and BAK into cytochrome c releasing pores. This process is governed by the BCL-2 protein family, whose pro-survival members (BCL-2, BCL-X_L_, MCL-1) suppress MOMP by sequestering pro-apoptotic effectors and BH3-only proteins, while BH3-only proteins (BIM, tBid, BAD) tip the balance toward cell death [126]. The founding observation linking VDAC to this pathway initially came from Shimizu’s lab, based on experiments with VDAC-containing proteoliposome, that BAX and BAK supposedly create a novel large pore with VDAC that allows cytochrome c passage [127]. Based on extensive work from the Shimizu lab, VDAC was hypothesized to be part of MOMP [128]. However, subsequent studies contradict this model (see review [129]), and Baines et al. showed that mouse embryonic fibroblast (MEF) cells can undergo apoptosis even in the absence of all three VDACs [130].

Recently, VDAC1 has been suggested to form a homo-oligomeric “mega-pore” that facilitates the release of cytochrome c and mitochondrial DNA, thereby inducing apoptosis in many diseases, such as lupus-like diseases [131], diabetes [132,133], and neurodegenerative diseases [134,135]. These studies extrapolate the presence of higher-molecular-weight bands in SDS-PAGE upon cross-linking to “mega-pore” formation. VDAC’s tendency to assemble into oligomeric structures in native mitochondrial membranes has been known since 1984 from pioneering EM work by Carmen Mannella’s group [136,137]. Later, VDAC’s ability to form higher-order complexes was confirmed by atomic force microscopy and cryo-EM [27,138,139,140]. Notably, VDAC1 oligomeric assemblies are sensitive to lipid composition, as shown on giant proteoliposomes with reconstituted VDAC1 by fluorescence correlation spectroscopy [141] and by atomic force microscopy [142] (see review by Di Pinto et al. [143] for a comprehensive discussion).

The loss of higher-molecular-weight bands upon treatment with the VDAC1 oligomerization inhibitor VBIT-4 has been routinely used to confirm the involvement of VDAC “mega-pores” in apoptosis and inflammation. However, it was recently shown that VBIT-4 does not interact with VDAC1 but may affect membrane proteins, especially mitochondrial proteins, via membrane modulation [144]. In fact, VBIT-4 was also shown to inhibit ETC complexes (I, III, and IV) and thus decrease mitochondrial potential and increase oxidative stress, leading to cell death [145]. In addition, VDAC oligomeric arrays may be important for rapid Ca^2+^ uptake from ER or SR. Therefore, changes in VDAC oligomerization can affect Ca^2+^ uptake, leading to oxidative stress and apoptosis without forming a “mega-pore”. Furthermore, the VDAC “mega-pore” has never been visualized, unlike BAK/BAX pores [146]. BAX/BAK have been shown to form proteolipid pores independently of VDAC [146,147,148], where VDAC serves as a context-dependent organizer or regulator of BCL-2 family proteins at the MOM.

VDAC, specifically VDAC1, has been reported to interact with BCL-X_L_ [149], promoting the open state of VDAC to sustain mitochondrial metabolite flux as a pro-survival bioenergetic function [18]. However, to date, the physiological relevance of direct VDAC1–BCL-2 family complexes and whether VDAC1 plays an indispensable role in apoptosis remain under debate.

Unlike VDAC1, VDAC2 has emerged as the isoform with the most clearly established role in apoptotic regulation. Mice KO studies showed that only VDAC2 KO led to embryonic lethality [19]. This was linked to the pro-survival function of VDAC2 due to its complexation with monomeric BAK, which is localized to MOM through its C-terminal transmembrane anchor [150]. Upon apoptotic stimulation, according to one model, BH3-only activators such as tBID displace VDAC2 from BAK, releasing BAK to oligomerize and permeabilize the MOM [151]. Using VDAC1-VDAC2 chimeric constructs in VDAC2 KO MEF cells, it was shown that the cytosolic loop between β-strands 10-11 of VDAC2 is a BAK docking site, with residues T168 and D170 being critical [21]. A small molecule, WEHI-9625, was shown to stabilize this interaction to prevent BAK-driven apoptosis [152]. Deep mutational scanning confirmed these findings and suggested that the cytosolic loop between β-strands 10-11 occupies the hydrophobic groove of BAK to stabilize the VDAC2-BAK complex [153]. Recent studies highlight VDAC2 as a mitochondrial checkpoint in cancer therapy through complexation with BAK [154,155,156]. Normal hepatocytes express low levels of both VDAC2 and BAK relative to hepatocellular carcinoma cells, rendering them resistant to VDAC2-directed pro-apoptotic interventions [157]. This raises a new challenging conundrum in the field: the possibility of distinct physiological roles for VDAC2 in normal versus cancer cells, further complicating the VDAC-involving signaling network. Furthermore, these studies emphasize the pharmacological disruption of the VDAC2–BAK interface as a novel therapeutic target [158].

In contrast to its pro-survival function through complexation with BAK, VDAC2 KO was reported to inhibit BAX apoptotic function, suggesting its pro-apoptotic role [22]. BAX is predominantly cytosolic due to sequestration of its C-terminal transmembrane domain in its hydrophobic groove [159]. VDAC2-BAX complexation has been suggested to facilitate BAX translocation to mitochondria upon apoptotic simulation [160]. However, BAX retained the ability to accumulate in MOM and mediate cell death upon apoptotic stimulation in VDAC2-deficient cells and required BAK for mitochondrial localization [161]. The conflicting results in the literature highlight the complex regulation of BAX recruitment [162] and require further investigation to clarify the role of VDAC2 in pro-apoptotic recruitment of BAX.

## 7. VDAC2-TOM Complex

VDAC is increasingly recognized as a functional partner of the TOM protein import machinery. Sakaue et al. found that yeast VDAC ortholog, porin (Por1), binds newly imported Tom22 and acts as a chaperone for unassembled Tom22, thereby regulating the balance between trimeric and dimeric TOM complexes and influencing mitochondrial protein import [163]. Takeda et al. confirmed that the Tom22-Por1 complex facilitates protein import into the IMS [140]. Ellenrieder et al. extended this concept by showing that Por1 also promotes carrier biogenesis after TOM-mediated entry, interacting with precursor proteins in the IMS and recruiting TIM22 to support transfer to the inner membrane [164].

**Figure 5:**
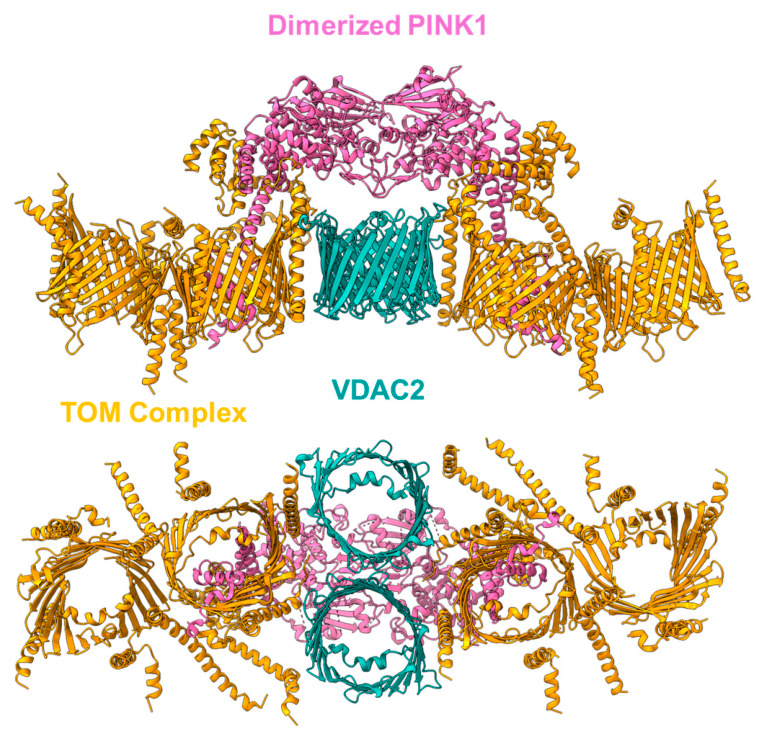
Cryo-EM structure of VDAC2 supercomplex with mitochondrial TOM complexes and cytosolic dimeric PINK1. Top: Cartoon representation of VDAC2 (teal), TOM complex (orange), and PINK1 (pink). Bottom: IMS view of the supercomplex derived from cryo-EM (PDB: 9EIH).

Most recently, Callegari et al. resolved a human TOM-VDAC2 array in which two TOM core complexes flank a central VDAC2 dimer to stabilize PINK1 at depolarized mitochondria, providing direct structural evidence that VDAC2 can be incorporated into higher-order TOM assemblies in mammalian cells [7] (Figure 5). Taken together, these recent studies support a model in which VDAC2 is not simply adjacent to TOM, but can function as a dynamic organizer and coupling factor for TOM-dependent protein import.

## 8. Other VDAC Complexes

VDAC has been found to interact with other proteins not discussed in this review, and new complexes are constantly being identified through recent advances in proteomics. In addition to the proteins discussed above, Table 1 lists other proteins suggested to interact with VDAC and regulate cellular processes such as metabolism (Mitochondrial creatine kinase and glycerol kinase), steroidogenesis (TSPO and StAR), and apoptosis (MCL1 and tBid). Many proteins linked to neurodegeneration have also been shown to interact with VDAC (Tau, Amyloid beta, Parkin, and MIRO-1). However, further studies are needed to confirm the direct complexation and regulation of VDAC by these proteins and their isoform specificity.

## 9. Summary

The simultaneous access of VDAC to otherwise strictly compartmentalized proteins on both sides of the MOM helps explain the growing interest in the physiological consequences of VDAC complex formation with its many cytosolic and mitochondrial partners. This emerging role as a cellular communication hub—distinct from, yet intrinsically linked to, its conventional function as a metabolite channel—explains how such a “simple” ß-barrel channel can contribute to a remarkably wide range of mitochondria-associated pathologies, including diabetes, cardiovascular diseases, various cancers, and neurodegenerative disorders.

**Figure 6:**
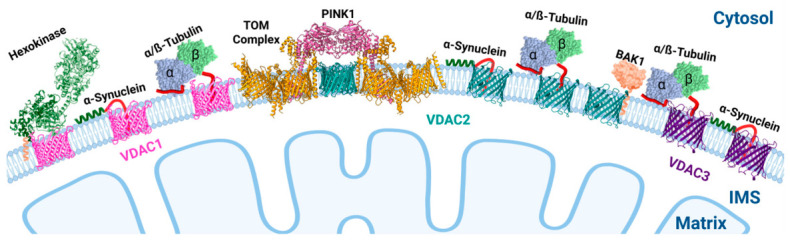
From passive holes to the cellular hub through complexation with cytosolic protein partners. The cartoon shows the array of cytosolic proteins reported to form complexes with all three VDAC isoforms. Created on BioRender.

Given the higher expression levels of VDAC1 in most cells and the availability of specific antibodies, it is the most well-characterized isoform. Many proteins have been identified as targeting VDAC1 by proteomics screening, and in a number of cases, further studies have confirmed the specificity of such binding and its impact on the channel function and cell physiology. VDAC2 has recently emerged as pivotal in regulating Ca^2+^ signaling and apoptosis in cardiac cells and cancer. VDAC3 interacts with αSyn and dimeric tubulin but with 10-100 times lower affinity than VDAC1 [96] (Figure 6). A proteomic study identified multiple VDAC3-interacting proteins [175]; however, the specificity of these interactions remains unconfirmed. Therefore, systematic isoform-resolved interactomics and structural studies to understand the molecular mechanisms of VDAC isoforms’ function and the identification of their natural regulators and synthetic inhibitors are vital for developing targeted therapies to improve outcomes in mitochondria-associated pathologies.

## Figures and Tables

**Table 1:** List of VDAC isoform-specific protein complexes.

	VDAC	VDAC1	VDAC2	VDAC3
**Metabolism**				
Hexokinase 1		✓ [49,51]	**~** [51]	**~** [52]
Hexokinase 2		✓ [47,48,49]	?	?
Mitochondrial Creatine Kinase		✓ [165]	?	?
Glycerol Kinase		✓ [27]	✓	✓
**Steroidogenesis**				
TSPO		✓ [166,167]	?	?
StAR		✓ [168]	✗ [168]	?
**TOM Complex**				
TOM5		?	✓ [7]	?
TOM20		?	✓ [7]	?
TOM22	✓ [140,163]	?	?	?
TIM22	✓ [164]	?	?	?
**MAM Complexes**				
IP3R-GRP75		✓ [103,105]	✓ [106]	?
Ryanodine Receptor 2		?	✓ [108]	?
CKAP4		✗	✓ [106]	✗
TMEM43		✓ [124,125]	✓ [124]	✗
Lamin B Receptor		✓	✓ [107]	✓
TRPML1 (Lysosome)		✓ [101]	✗	✗
PhosphoInositol-3-Kinase (Endosome)		?	✓ [169]	?
**BCL-2 Family**				
BCL-X_L_		✓ [170]	**~** [160]	✓ [170]
MCL1		✓ [171]	✗	✓ [171]
BAK		✗	✓ [19]	✗
BAX		**~** [160]	✓ [22]	✗
tBid	✓ [20]	?	?	?
**Cytoskeleton**				
Dimeric Tubulin		✓ [30]	✓ [Unpublished]	✓ [96]
Actin	✓ [29]	?	?	?
Desmin	✓ [172]	?	?	?
**Neurodegeneration**				
α-Synuclein		✓ [32]	✓ [97]	✓ [96]
Phosphorylated Tau		✓ [34]	?	?
Amyloid Beta		✓ [34]	?	?
Parkin		✓ [173]	✓	✓
MIRO-1		✓ [174]	✗	✗

✓ Yes; ✗ No; ~ Debated; ? Unknown.

## Data Availability

No new data were created or analyzed in this study. Data sharing is not applicable to this article.

## Abbreviations

The following abbreviations are used in this manuscript:

VDAC	Voltage-Dependent Anion Channel
MOM	Mitochondrial outer membrane
IMS	Intermembrane space
HK	Hexokinase
αSyn	Alpha-synuclein
KO	Knockout
MBD	Mitochondria Binding Domain
CTT	C-Terminal Tail
PLM	Planar lipid membrane
MD	Molecular Dynamics
PLA	Proximity ligation assay
ER	Endoplasmic Reticulum
SR	Sarcoplasmic Reticulum
MAM	Mitochondria associated membranes
ERAD	ER-associated degradation
UPR	Unfolded Protein Response
PD	Parkinson’s Disease
MOMP	MOM permeabilization
